# Impact of transcranial direct current stimulation on white matter microstructure integrity in mild cognitive impairment patients according to effect modifiers as risk factors for Alzheimer’s disease

**DOI:** 10.3389/fnagi.2023.1234086

**Published:** 2023-09-01

**Authors:** Dong Woo Kang, Sheng-Min Wang, Yoo Hyun Um, Sunghwan Kim, TaeYeong Kim, Donghyeon Kim, Chang Uk Lee, Hyun Kook Lim

**Affiliations:** ^1^Department of Psychiatry, Seoul St. Mary’s Hospital, College of Medicine, The Catholic University of Korea, Seoul, Republic of Korea; ^2^Department of Psychiatry, Yeouido St. Mary’s Hospital, College of Medicine, The Catholic University of Korea, Seoul, Republic of Korea; ^3^Department of Psychiatry, St. Vincent’s Hospital, College of Medicine, The Catholic University of Korea, Seoul, Republic of Korea; ^4^Research Institute, NEUROPHET Inc., Seoul, Republic of Korea

**Keywords:** transcranial direct current stimulation, amyloid-beta deposition, *APOE* ε4 allele, BDNF Val66Met polymorphism, sex

## Abstract

**Background:**

Little research exists on how individual risk factors for Alzheimer’s disease (AD) affect the intermediate phenotype after transcranial direct current stimulation (tDCS), despite the importance of precision medicine-based therapeutic approaches.

**Objective:**

To determine how an application of sequential tDCS (2 mA/day, left dorsolateral prefrontal cortex, 10 sessions) affects changes in white matter (WM) microstructure integrity in 63 mild cognitive impairment (MCI) patients with effect modifiers such as Aβ deposition, *APOE* ε4 carrier status, BDNF Val66Met polymorphism status, and sex.

**Methods:**

We examined individual effect modifier-by-tDCS interactions and multiple effect modifiers-by-tDCS interactions for diffusion metrics. We also evaluated the association between baseline Aβ deposition and changes in WM microstructure integrity following tDCS.

**Results:**

We found that *APOE* ε4 carrier status and sex had a significant interaction with tDCS, resulting in increased fractional anisotropy (FA) in the right uncinate fasciculus (UF) after stimulation. Additionally, we observed multiple effect modifiers-by-tDCS interactions on WM integrity of the right UF, leading to a more pronounced increase in FA values in *APOE* ε4 carriers and females with Val66 homozygotes. Finally, baseline Aβ deposition was positively associated with a difference in FA of the left cingulum in the hippocampal area, which showed a positive association with the changes in the score for delayed memory.

**Conclusion:**

Our study shows the differential impact of individual AD risk factors on changes in the early intermediate phenotype after sequential tDCS in MCI patients. This research emphasizes the importance of precision medicine approaches in tDCS for the prodromal stages of AD.

## Introduction

Alzheimer’s disease (AD) is a representative neurodegenerative disorder characterized by the deposition of amyloid beta (Aβ) and tau proteins, leading to impairment of cognitive function and the ability to perform daily activities (Scheltens et al., 2016). Mild cognitive impairment (MCI) is a prodromal stage of AD and approximately 10–15% of patients with MCI have been reported to convert to dementia each year (Gauthier et al., 2006). Despite the high risk of progression to dementia, treatment options for MCI are limited. Although several drugs have been investigated in clinical trials, their efficacy in delaying or preventing progression to dementia is modest and uncertain (Anderson et al., 2017). Therapeutic approaches such as cognitive intervention (Jean et al., 2010), physical exercise (Lautenschlager et al., 2010), and dietary modification have demonstrated some promising outcomes in terms of changes in cognitive function and biomarkers (Singh et al., 2014). However, further research is required to establish these interventions as effective strategies for preventing AD. Additionally, the complexity of these interventions can make them challenging for individuals with MCI to implement and maintain consistently (Coley et al., 2019), highlighting the need for a simple, fixed intervention that can be sustained over a specific period. In this regard, there is currently a need for alternative interventions that are accessible to individuals with MCI and can improve cognitive function and mitigate neurodegenerative changes. An interest in non-invasive brain stimulation treatment methods is also increasing, and in particular, transcranial direct current stimulation (tDCS), which is highly accessible in terms of cost and portability, is proposed as one of the appropriate treatment options for MCI (Liu et al., 2017). tDCS modulates the excitability of cortical neurons depending on the current flow direction and has synaptic after-effects through long-term potentiation (LTP), affecting neuroplasticity (Nitsche et al., 2003). It is also suggested to normalize brain function in patients with AD and facilitate the clearance of Aβ by modulating the integrity of the brain–blood barrier (Meinzer et al., 2015; Shin et al., 2020). Previous studies have shown that cognitive function can be improved through single or multi-session tDCS in patients with AD and MCI (Lu et al., 2019; Manenti et al., 2020). Moreover, long-term application of this technique is expected to modulate disease progression (Im et al., 2019; Yang et al., 2019).

Among intermediate phenotypes related to AD, changes in white matter microstructure have been demonstrated to occur earlier in dementia than changes in brain function, making it a useful early warning sign for the development of the disease (Zhang et al., 2009). Additionally, advanced imaging techniques, such as diffusion tensor imaging (DTI), can be used to quantify white matter assessment, allowing for more precise and objective measurement of changes over time (Le Bihan and Johansen-Berg, 2012). Patients with MCI have shown a reduced fractional anisotropy (FA) value, which reflects white matter integrity, in multiple posterior white matter regions, as well as in frontal, temporal, parietal, and occipital white matter and association fibers compared to normal subjects (Medina et al., 2006). Moreover, lower FA values of the splenium of corpus callosum and crus of fornix have accurately differentiated between amnestic MCI patients and control subjects (Medina et al., 2006). Furthermore, the deterioration of white matter microstructure has predicted a more rapid transition to MCI in cognitively intact older adults (Shafer et al., 2022). Additionally, prior research has indicated that the white matter microstructure changes in a non-linear manner as the clinical phenotype progresses and the extent of Aβ deposition increases (Dong et al., 2020; Pereira et al., 2021). In this regard, a connection to compensatory mechanisms has been proposed (Raghavan et al., 2021).

Despite the clinical evidence of white matter microstructure as an intermediate phenotype in the prodromal AD phase, few studies have examined changes in white matter microstructure after tDCS in MCI patients. In other vascular and neurodegenerative diseases, previous studies have shown that FA values increase in the frontal lobe after tDCS application in stroke patients with memory impairment (Hua et al., 2022). The white matter integrity of ventral language pathways has also predicted letter accuracy in primary progressive aphasia after tDCS application (Zhao et al., 2021).

Precision medicine, which involves personalized strategies based on an individual’s genetic, biomarker, and clinical profile, has gained traction as a promising approach in the management of AD (Isaacson et al., 2018). It can help optimize tDCS treatment response and lead to more effective and efficient AD management by considering individual AD risk factors. Aβ deposition has been shown to affect the rate of cognitive decline and intermediate phenotype of brain structure and function, as well as the risk of transitioning to dementia in patients with MCI (Lukiw et al., 2020). In addition, *APOE* ε4 allele has been shown to affect the likelihood and severity of the Aβ pathophysiological cascade and is responsible for the greatest proportion of genetic risk factors for sporadic AD (Harrison et al., 2020). Brain-derived neurotrophic factor (BDNF) is a protein that impacts the growth and maintenance of neurons and has been known to play an important role in LTP-like neuroplasticity induced by tDCS (Chaieb et al., 2014). Previous studies have shown that its levels are decreased in AD (Ng et al., 2019). Moreover, research has suggested that BDNF Val66Met polymorphisms may modulate the risk of AD by affecting BDNF levels (Du et al., 2018). In regard to sex, research suggests that there are distinct differences in the clinical and intermediate phenotype patterns of males and females with AD (Ferretti et al., 2018). Specifically, studies have shown that females with MCI and dementia tend to experience a more rapid cognitive decline and brain atrophy after diagnosis (Ferretti et al., 2018). However, there have been few studies examining the impact of tDCS treatment based on individual AD risk factors, which would allow for more precise medical treatment strategies using tDCS in the prodromal stage of AD. In our previous pilot study in MCI patients, we have found that brain functional integration and segregation parameters differ after sequential tDCS to the left dorsolateral prefrontal cortex (DLPFC) depending on Aβ deposition and *APOE* ε4 carrier status (Kang et al., 2021). The BDNF Val66Met polymorphism has been reported to have a duration-dependent effect on tDCS-induced motor cortex plasticity in older adults (Puri et al., 2015). However, other studies have exhibited inconsistent results on the impact of BDNF Val66Met polymorphism on cortical excitability (Antal et al., 2010; Teo et al., 2014). Regarding sex, previous research has reported a differential impact of tDCS on practice-related executive function in older adults, with higher current density observed in female older adults (Fehring et al., 2021). Therefore, it is worthwhile to investigate how the impact of tDCS on white matter microstructure integrity in the AD prodromal phase is modified by representative individual AD risk factors as effect modifiers.

In this context, we aimed to evaluate whether a 2-week application of sequential tDCS alters white matter microstructure integrity in patients with MCI and whether it depends on effect modifiers consisting of individual risk factors for AD. Furthermore, for white matter microstructure tracts that exhibit a significant association with baseline Aβ deposition, we assessed the correlation with the differences in cognitive function scores to elucidate the nature of the alteration.

## Materials and methods

### Participants

Participants were recruited through paper postings at the Brain Health Center, Yeoui-do St. Mary’s Hospital, College of Medicine, the Catholic University of Korea, Republic of Korea. Inclusion criteria included the following: (1) participants who met Peterson’s criteria for MCI (Petersen, 2004); (2) a Clinical Dementia Rating (CDR) score of 0.5. Potential participants were excluded for the following: (1) a history of alcoholism, drug abuse, head trauma, or psychiatric disorders; (2) taking any psychotropic medications (e.g., cholinesterase inhibitors, antidepressants, benzodiazepines, and antipsychotics); (3) contraindications to receiving tDCS or undergoing a MRI scan (ferromagnetic or coiled metal implants); (4) any skin disorder that compromised skin integrity over the scalp. The assessment process for inclusion and exclusion criteria was conducted by two geriatric psychiatrists. All potential participants consented to medical chart reviews. Additionally, all assessments were performed at the Brain Health Center, Yeoui-do St. Mary’s Hospital, College of Medicine, the Catholic University of Korea, Republic of Korea. Study procedures were conducted in accordance with the Declaration of Helsinki and was approved by the Institutional Review Board of the Catholic University of Korea (SC19DEST0012). Informed and written consent was obtained from all participants. This study is registered with the Clinical Research Information Service of Korea Disease Control and Prevention Agency (KCT0006020). The study was conducted from May 2020 to February 2022, and all on-site study procedures were performed at the Brain Health Center, Yeoui-do St. Mary’s Hospital, College of Medicine, the Catholic University of Korea, Republic of Korea. The investigators have no ethical or financial conflict of interests with respect to the manufacturers of any of the equipment used in the study.

### Study protocol

**Figure 1:**
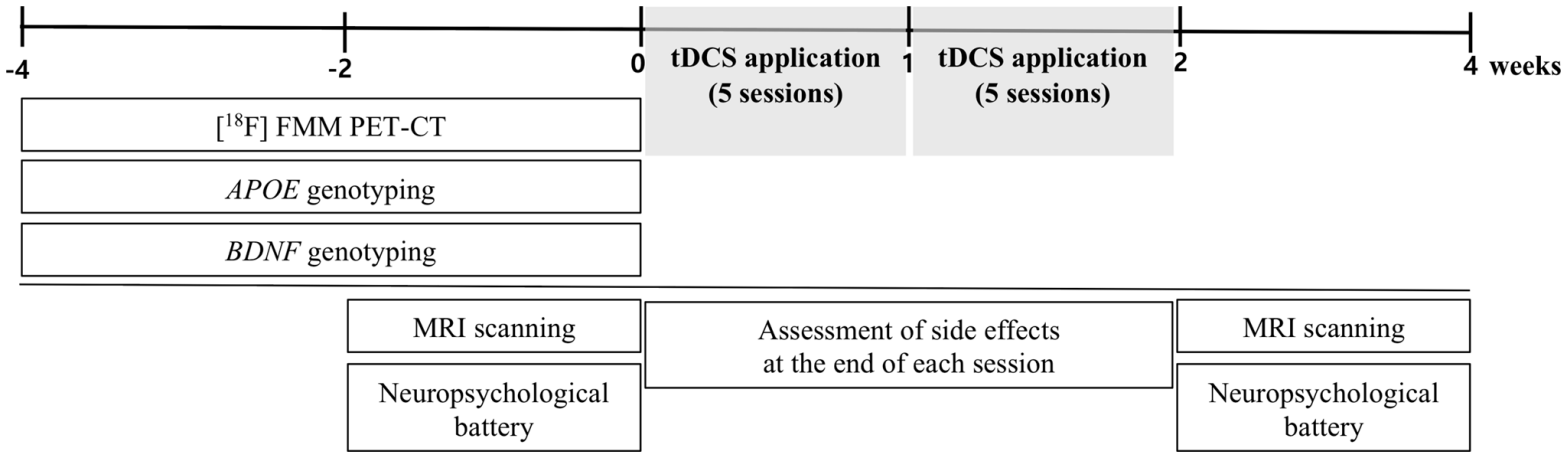
Schematic diagram showing timeline of experimental procedures. FMM, flutemetamol; BDNF, Brain-derived neurotrophic factor.

This study was conducted as a single-arm prospective trial, without the use of a sham condition. In this study, patients received 10 tDCS sessions at the patient’s residence (five times per week for 2 weeks, totaling 10 sessions). We selected 10 sessions based on the results of previous clinical studies that used 10 sessions of tDCS to treat patients with AD and MCI (Cotelli et al., 2014; Khedr et al., 2014; Manenti et al., 2016; Roncero et al., 2017). We also considered the treatment adherence of older patients. The participants were assessed with a neuropsychological battery and underwent MRI scanning within 2 weeks before the first tDCS session and after the 10th session at the Brain Health Center of Yeouido St. Mary’s Hospital. Subjects also underwent [^18^F] flutemetamol (FMM) positron emission tomography–computed tomography (PET-CT), as well as *APOE*, and BDNF genotyping within 4 weeks before the first tDCS session. We have assessed side effects using the Udvalg for Kliniske Undersogelser side-effect rating scale at the end of each session (Lambert et al., 2003). In addition, participants and the psychologists who performed the neuropsychological battery were blinded to the results of amyloid-PET, *APOE*, and BDNF genotyping. Figure 1 displays a schematic diagram that illustrates the experimental procedures.

### Transcranial direct current stimulation application

A constant direct current (2 mA, 20 min) was administered by an MRI-compatible stimulator (YDS-301 N, YBrain, Seoul, Republic of Korea). The anode was attached over the left DLPFC (F3 in the International 10/20 electroencephalogram system). The cathode was positioned over the right supraorbital region. The electrodes touched a saline-soaked sponge (disk type, radius = 3 cm) placed on the scalp. The staff skilled in the use of the device visited the patient’s residence for each stimulus session to guide device application. To ensure that the electrodes were placed in the same location throughout the 10 stimulation sessions per participant, the staff used the international 10/20 electroencephalogram system, as well as electrode center locations relative to anatomical landmarks (nasion, inion, left and right preauricular points and vertex) identified on the participants’ faces and heads. A vertex is defined as the intersection of the line connecting the nasion and inion and the line connecting both preauricular points. Position one end of a tape measure starting at one preauricular point and passing through the center of the electrode and note the intersection of that line with the line from the vertex to nasion. We also recorded the distance from the bilateral preauricular point to the center of the electrode and checked its position and distance to landmarks before the start of each session. Finally, to ensure the accuracy of the positioning, the staff double-checked the position of the electrodes 15 min after the start of the session. Additionally, the staff for each participant was kept unchanged throughout the 10 sessions.

### Neuropsychological assessment

Cognitive function was assessed in all subjects using the Korean version of the Consortium to Establish a Registry for AD (CERAD-K; Lee et al., 2002). The measurements included assessments of the Korean version of the verbal fluency (VF) test, the 15-item Boston Naming Test, Mini-Mental State Examination (MMSE-K; Park, 1989), word list memory (WLM), word list recall (WLR), word list recognition (WLRc), constructional praxis, and constructional recall. In addition, total memory (TM) scores were obtained by summing the respective scores from the WLM, WLR, and WLRc tests. The total CERAD-K scores were calculated by summing all subcategory scores, excluding the MMSE-K and constructional recall cores. Higher Trail Making Test B scores indicate lower executive function. Details regarding the use of specific tests and the reviewing process are described in the Supplementary Material.

### Processing procedures of the DTI images

The procedures for magnetic resonance imaging acquisition are described in the Supplementary Material. The data was preprocessed using Statistical Parametric Mapping 12 (SPM12)^1^ running on MATLAB version 2018b, the PANDA toolbox,^2^ and the FMRIB software library v6.0.^3^ The main procedure of PANDA includes (1) preprocessing; (2) producing diffusion metrics. The workflow of preprocessing includes five steps: (1) estimating the brain mask; (2) cropping the raw images; (3) correcting for the eddy-current effect; (4) averaging multiple acquisitions; and (5) calculating diffusion tensor (DT) metrics. We used FA, mean diffusivity (MD), and RD (radial diffusivity) as the DT metrics. Then, the images of the diffusion metrics were normalized to the MNI standard space for further analysis. For diffusion metrics images with voxel size of 1.0 × 1.0 × 1.0 mm^3^ in the standard space, the regional averages were calculated according to prior white matter tract atlas (WM probtract atlas). This atlas comprises 20 regions, which are identified probabilistically by averaging the results of running deterministic tractography on 28 normal subjects (Hua et al., 2008). The WM probtract atlas includes the following WM tracts; (1) anterior thalamic radiation; (2) cingulum in the cingulate cortex area; (3) cingulum in the hippocampal area; (4) corticospinal tract; (5) forceps major; (6) forceps minor; (7) inferior fronto-occipital fasciculus; (8) superior longitudinal fasciculus (SLF); (9) the temporal projection of the SLF; (10) inferior longitudinal fasciculus; (11) uncinate fasciculus (UF). We obtained statistics result files for each WM tract in the left and right hemisphere (excluding forceps major and minor). These files contain several diffusion measures, including FA, MD, and RD. Each DT metric reflects a different aspect of white matter integrity (Mori and Zhang, 2006). (1) FA: This parameter represents the degree of anisotropy or directionality of water molecule diffusion within a voxel. Higher FA values usually signify greater directionality and are indicative of better white matter microstructure integrity; (2) MD: MD is a measure of the overall magnitude of water diffusion, irrespective of direction, within a voxel. Higher MD values typically indicate increased overall water movement, which can be suggestive of less dense white matter, potential injury, or degradation; (3) RD: RD is a measure of water diffusion perpendicular to the principal direction of diffusion. RD is often considered as a potential index of myelin integrity, with higher RD values interpreted as representing potential demyelination. Details regarding the main procedure of PANDA are described in the previous study (Cui et al., 2013).

### *APOE* genotyping

The procedures for *APOE* genotyping are described in the Supplementary Material. Considering the protective effect of *APOE* ε2 allele (Li et al., 2020), we excluded participants with the *APOE* ε2 allele. If a participant had at least one *APOE* ε4 allele, they were categorized as an *APOE* ε4 carrier; if they had no *APOE* ε4 allele, they were categorized as an *APOE* ε4 non-carrier.

### BDNF genotyping

The procedures for BDNF genotyping are described in the Supplementary Material. For BDNF Val66Met polymorphism (rs6265), the genotype groups were divided into Met66 allele carrier and Met66 allele non-carrier (Val66 homozygote) groups according to previous genetic studies on this genotype (Carballedo et al., 2012; Han et al., 2015). If a participant had at least one Met66 allele, they were categorized as a Met carrier; if they had no Met66 allele, they were categorized as a Met non-carrier.

### SUVR calculation

Information on PET scanners and the procedures for [^18^F]-FMM PET image acquisition and processing are described in the Supplementary Material. The semi-quantification of [^18^F] FMM uptake on PET/CT scan was performed by obtaining the standardized uptake value ratios (SUVRs). The volumes of interest (VOIs) were restricted to gray matter, covering the frontal, superior parietal, lateral temporal, anterior, and posterior cingulate cortex/precuneus regions. These VOIs are known to be preferentially affected by Aβ deposition in the early stages of AD (Thal et al., 2002) and were also considered in a previous study (Thurfjell et al., 2014). The reference region for SUVR calculations was pons. The pons does not typically show significant Aβ deposits even in later stages of AD (Thal et al., 2002) and has been utilized as a reference area for SUVR measurements (Landau et al., 2014). The mean uptake counts of each VOIs and reference region were measured on the preprocessed image. A regional SUVR was calculated as the ratio of each cortical regional mean count to the pons mean count (SUVR_PONS_). The global cortical average (composite SUVR) was calculated by averaging regional cortical SUVRs weighted for size. We used a cut-off of 0.62 for “positive” versus ‘negative’ neocortical SUVR, consistent with the cut-off values used in a previous [^18^F] FMM PET study (Thurfjell et al., 2014). PET scans classified with negative Aβ accumulation also exhibited normal visual reading.

### Statistical analysis

Statistical analyzes were performed using R software (version 2.15.3), jamovi (version 1.6.23),^4^ and SPM 12. Assumptions of normality were tested for continuous variables using the Kolmogorov–Smirnov test in R software; all data demonstrated a normal distribution and were standardized by z-score transformation for the analysis.

Repeated-measures ANOVA was used to predict the impact of effect modifier-by-tDCS interaction (effect modifier*tDCS) for diffusion metrics (i.e., FA, MD, and RD), with tDCS (pre- and post-tDCS) as repeated-measures factor and *APOE* ε4 carrier status, BDNF Val66Met polymorphism status, and sex as the between-subject factor (effect modifier), with covariates of age, years of education, and global [^18^F] FMM SUVR_PONS_. Using four between-subject factors did not allow for a suitable repeated-measures ANOVA. To address this issue, we evaluated Aβ deposition using global [^18^F] FMM SUVR_PONS_, which provides a continuous variable for Aβ deposition. This allowed us to reduce the number of between-subject factors. We examined not only individual effect modifier*tDCS interaction, but also multiple effect modifiers*tDCS interaction (effect modifier1*effect modifier2*tDCS) for diffusion metrics. With respect to Aβ deposition, we evaluated global [^18^F] FMM SUVR_PONS_*tDCS interaction.

In addition, partial correlation analysis was performed to evaluate the association between baseline [^18^F] FMM SUVR_PONS_ and differences in diffusion metrics (i.e., FA, MD, and RD), adjusting for age, sex, education years, *APOE* ε4 carrier, and BDNF Val66Met polymorphism status. For the differences in diffusion metrics of WM probtract atlas displaying a significant association with [^18^F] FMM SUVR_PONS_, we evaluated a relationship with a difference in neuropsychological performances by partial correlation analysis, adjusting for age, sex, education years, Aβ deposition, *APOE* ε4 carrier, and BDNF Val66Met polymorphism status. Furthermore, we explored the association between baseline [^18^F] FMM SUVR_PONS_ and differences in diffusion metrics in each subgroup stratified by AD risk factors, adjusting for age, education years, and AD risk factors except for Aβ deposition, and the other AD risk factors we used to stratify subgroups. For the differences in diffusion metrics of WM probtract atlas displaying a significant association with [^18^F] FMM SUVR_PONS_ in each subgroup, we also examined an association with a difference in neuropsychological performances by partial correlation analysis, adjusting for age, education years, and AD risk factors except for the risk factors we used to stratify subgroups. All statistical analyzes used a two-tailed *p*-value <0.05 to define statistical significance. For the results of partial correlation analyzes in subgroups stratified by AD risk factors, a *p*-value <0.01 was considered statistically significant given the small sample size.

## Results

### Baseline demographic and clinical data

**Figure 2:**
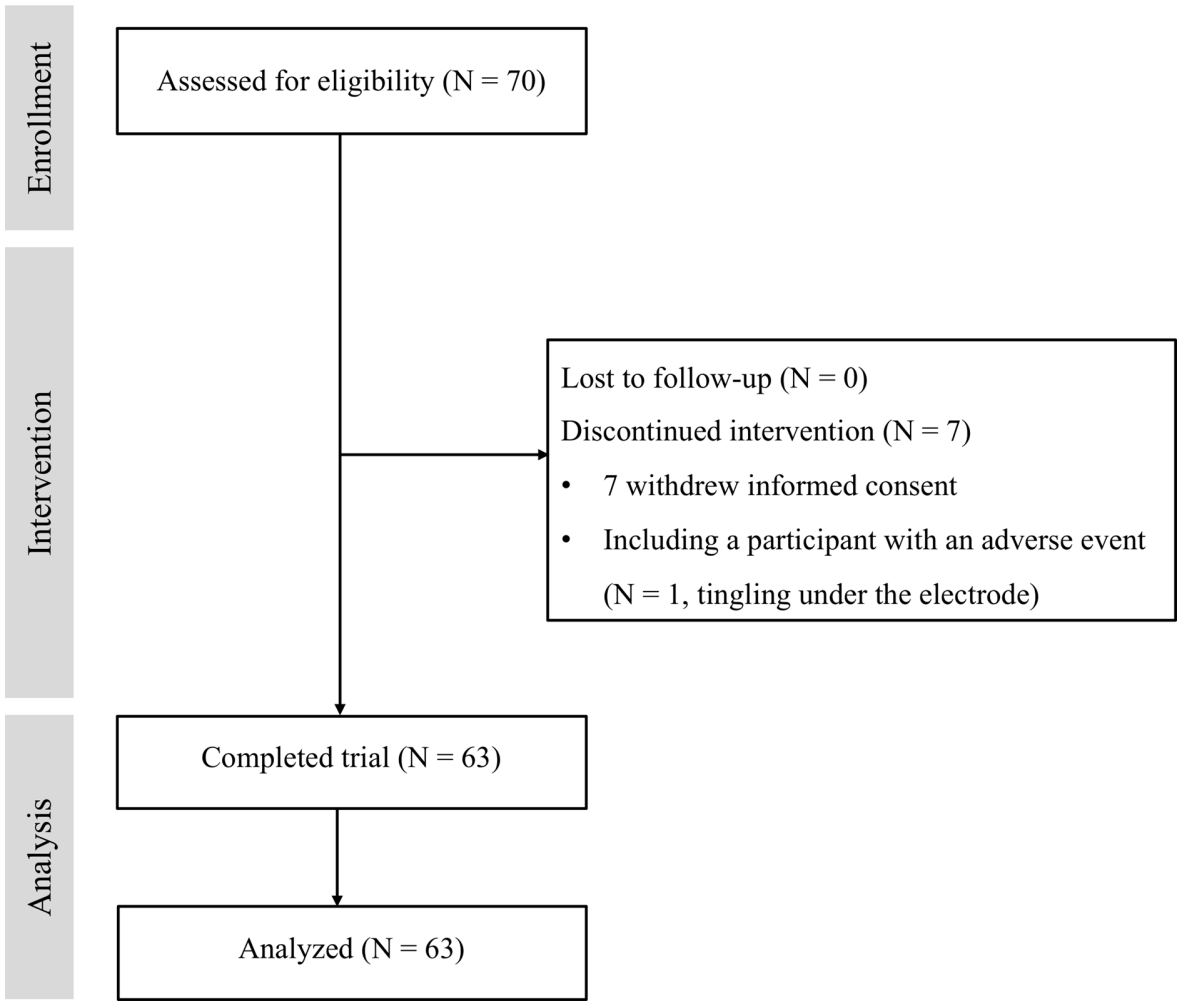
The flowchart of the study.

A total of 70 participants who met the inclusion and exclusion criteria were enrolled. Seven participants dropped out of the study due to refusal (*N* = 6) and a tDCS-related adverse event (*N* = 1, tingling under the electrode). Sixty-three subjects completed the study and were included in the analysis (Figure 2). Table 1 shows the baseline demographic data for the participants who completed the study.

**Table 1:** Baseline demographic and clinical characteristics of the study participants.

Demographic and clinical characteristics (*N* = 63)	
Age (years)	73.2 ± 7.9
Gender	
- Male	21 (33.3%)
- Female	42 (66.7%)
Years of education	12.0 ± 5.0
[^18^F] Flutemetamol deposition (positivity, %)	26 (41.3%)
Global [^18^F] Flutemetamol SUVR_PONS_	0.62 ± 0.15
*APOE* ε4 carrier status (carrier. %)	30 (47.6%)
BDNF polymorphism (Val/Met or Met/Met, %)	52 (82.5%)
CERAD-K	
VF	12.1 ± 5.1
BNT	10.7 ± 3.1
MMSE	23.2 ± 5.0
WLM	14.6 ± 4.6
CP	10.1 ± 1.5
WLR	3.7 ± 2.6
WLRc	6.8 ± 2.8
CR	4.5 ± 3.4
TMT B (seconds)	224.1 ± 77.7
Stroop word-color	26.0 ± 14.2
Total memory	25.2 ± 8.8
Total CERAD-K	58.1 ± 15.2

Data are presented as the mean ± SD unless indicated otherwise. SUVR_PONS_, standardized uptake value ratio of [^18^F] Flutemetamol, using the pons as a reference region; CERAD-K, Korean version of Consortium to Establish a Registry for Alzheimer’s Disease; VF, verbal fluency; BNT, Boston Naming Test; MMSE, the Korean version of the Mini-Mental Status Examination; WLM, Word List Memory; CP, Constructional Praxis; WLR, Word List Recall; WLRc, Word List Recognition; CR, constructional recall; TMT B, Trail Making Test B; Total memory, composite score summing scores of the WLM, WLR, and WLRc tests; Total CERAD-K, composite score summing scores of the CERAD-K VF, BNT, WLM, CP, WLR, and WLRc domains.

### Changes in white-matter microstructure integrity according to effect modifiers

**Figure 3:**
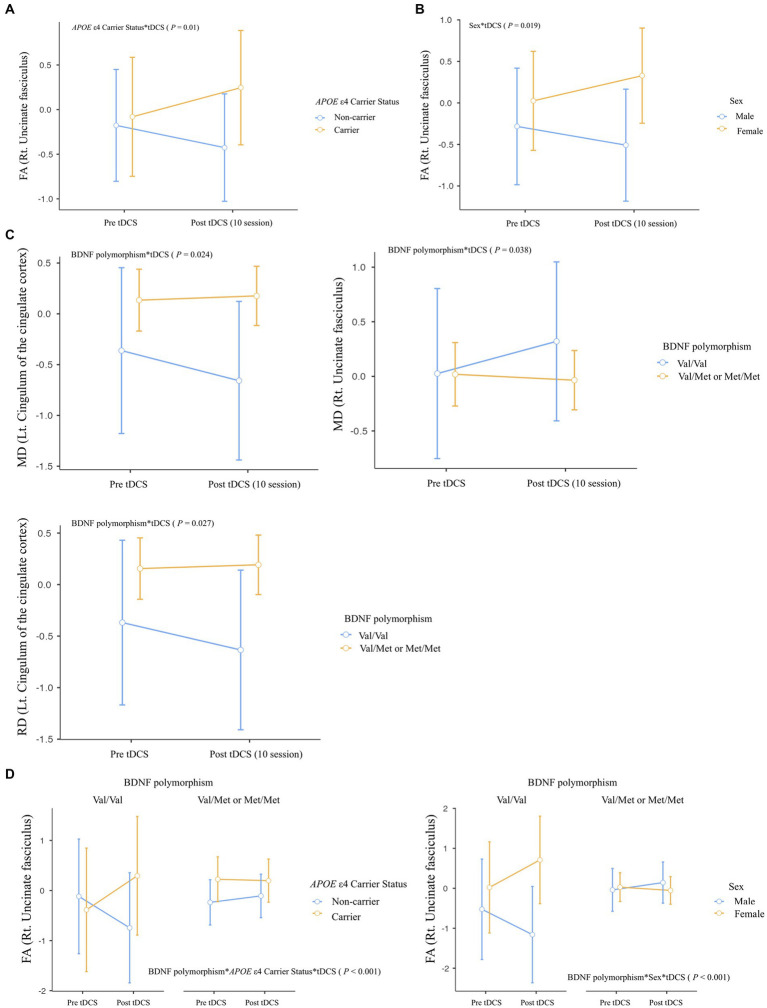
Differential impact of tDCS on white matter microstructural integrity according to effect modifiers: **(A)** *APOE* ε4 carrier status, **(B)** Sex, **(C)** BDNF polymorphism, and **(D)** Multiple effect modifiers. Repeated-measures ANOVA was used to predict the impact of effect modifier-by-tDCS interaction (effect modifier*tDCS) for white matter microstructural integrity (Between-subject factors: *APOE* ε4 carrier status, BDNF polymorphism, and sex), with covariates of age, years of education, and global [^18^F] FMM SUVR_PONS_. FA, fractional anisotropy; MD, mean diffusivity; RD, radial diffusivity.

We observed several important interactions in our study. First, a significant interaction was found between *APOE* ε4 carrier status and tDCS, manifesting as increased FA in the right UF (Rt. UF) of the *APOE* ε4 carriers (*p* = 0.01; Figure 3A). Moreover, a sex*tDCS interaction was also identified, which was shown by an increased FA in the Rt. UF of female participants (*p* = 0.019; Figure 3B). Interestingly, our study revealed a BDNF polymorphism*tDCS interaction. This was evidenced by decreased MD and RD in the left cingulum of the cingulate cortex and increased MD in the Rt. UF of the Met non-carriers (MD in Lt. cingulum of the cingulate cortex, *p* = 0.024; RD in Lt. cingulum of the cingulate cortex, *p* = 0.027; MD in Rt. UF, *p* = 0.038; Figure 3C).

Regarding multiple effect modifiers, our results showed a BDNF polymorphism*APOE ε4 carrier status*tDCS interaction, this was seen as increased FA in Rt. UF of Met non-carriers with *APOE* ε4 carrier status (*p* < 0.001; Figure 3D). Furthermore, a BDNF polymorphism*Sex*tDCS interaction was found, contributing to increased FA in Rt. UF of female participants with Met non-carrier status (*p* < 0.001; Figure 3D).

Despite these significant findings, no interaction between Aβ deposition and tDCS was observed for any of the diffusion metrics of each WM tract. A table containing all statistical comparisons shown in Figure 3 can be found in the Supplementary Results.

### Association between baseline Aβ deposition and changes in white matter microstructure integrity

**Figure 4:**
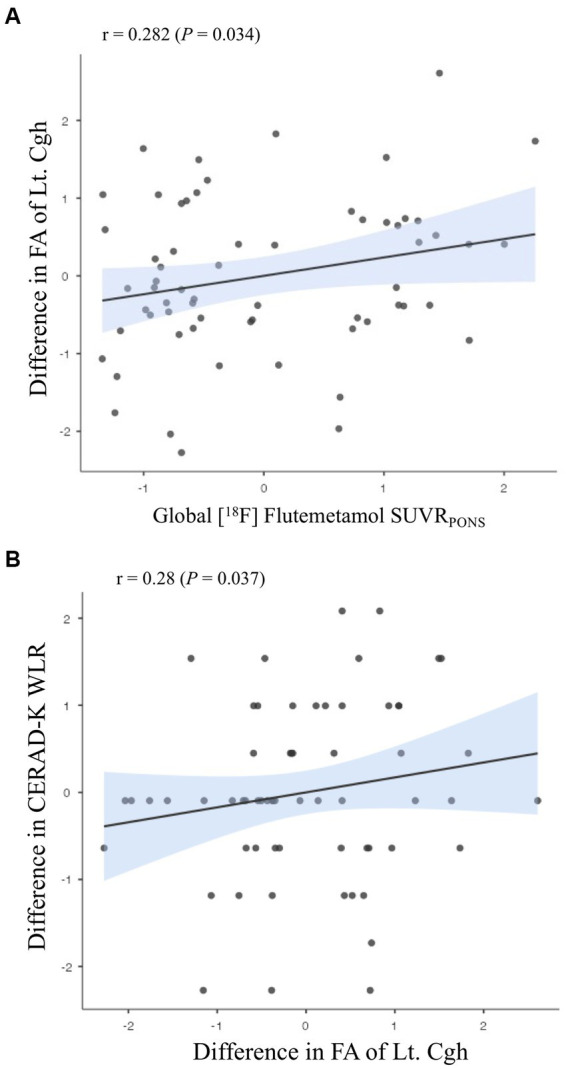
Associations **(A)** between amyloid-beta deposition and changes in white matter microstructural integrity; **(B)** between the differences in white matter microstructural integrity and those in neuropsychological performances. Partial correlation analysis adjusting for **(A)** age, sex, education years, *APOE* ε4 carrier status, and BDNF polymorphism; **(B)** age, sex, education years, Aβ deposition, *APOE* ε4 carrier status, and BDNF polymorphism. WLR, Word List Recall; FA, fractional anisotropy; Cgh, cingulum in the hippocampal area.

Concerning the relationship of difference in diffusion metrics with baseline Aβ deposition, there was a positive association in difference in FA of Lt. cingulum in the hippocampal area (Figure 4A; *r* = 0.282, *r*^2^ = 0.08, *p* = 0.034), which showed a positive relationship with a difference in CERAD-K WLR (Figure 4B; *r* = 0.28, *r*^2^ = 0.08, *p* = 0.037). Additionally, we found a positive association between Aβ deposition and the difference in FA of the left cingulum in the hippocampal area, which was observed not only in the overall group but also in *APOE* ε4 carriers (*r* = 0.518, *r*^2^ = 0.268, *p* = 0.007). Table showing the association between Aβ deposition and changes in white matter microstructural integrity in *APOE* ε4 carrier is provided in the Supplementary Results. However, for the difference in FA of this track of interest, there was not a significant association with a difference in neuropsychological performances in the *APOE* ε4 carriers. In subgroups excluding *APOE* ε4 carriers, we could not identify a significant association between baseline Aβ deposition and differences in diffusion metrics.

### Safety and tolerability

Among the enrolled 70 participants, one subject complained of tingling under the anode in the 4th session and withdrew informed consent (Figure 2). After dropping out, the subject reported improvement in tingling and recovered.

## Discussion

The present study was designed to investigate how the effect of 2 weeks of tDCS on white matter microstructural integrity in MCI patients varies according to an effect modifier composed of individual factors of AD, including Aβ deposition, *APOE* ε4 carrier status, BDNF Val66Met polymorphism status, and sex.

This study found a significant interaction between AD risk factors and tDCS for white matter microstructural integrity in tracts at risk for AD. The interaction between *APOE* ε4 carrier status and tDCS was attributed to increased FA in Rt. UF of *APOE* ε4 carriers after sequential tDCS sessions. The UF is a white matter tract involved in cognitive functions such as episodic memory and language (Von Der Heide et al., 2013), and previous studies have shown that FA levels in the UF decrease with the progression of AD (Liu et al., 2011; Qin et al., 2021). *APOE* ε4 carrier status has been found to differentially affect the integrity of white matter microstructure depending on age and severity of AD (Kljajevic et al., 2014), inducing better integrity of the UF compared with *APOE* ε4 non-carrier without a family history of AD (Adluru et al., 2014). However, there is a paucity of research on the effects of tDCS on white matter integrity at any stage of AD, including MCI, and there is also a lack of research on potential interactions with effect modifiers such as *APOE* ε4 carrier status. Taken together, the increased white matter microstructural integrity of the UF observed in *APOE* ε4 carriers after 10 sessions of sequential tDCS may reflect a compensatory mechanism for AD progression. However, given that white matter integrity is an intermediate phenotype for AD progression, further work is needed to clarify that changes in this metric are compensatory for the cognitive and functional deterioration. Given that the transition from MCI to AD takes 4–5 years (Vermunt et al., 2019), it is important to assess the impact of long-term changes in white matter microstructural integrity on changes in cognitive function and the risk of transition from MCI to AD.

In the present study, sequential tDCS led to increased FA in the Rt. UF of female subjects due to the sex*tDCS interaction. This may be due to the fact that white matter microstructure deterioration in females begins later and progresses more gradually than in males (Toschi et al., 2020), and older females receive a higher intensity of tDCS current at the target site compared to males, which may be due to the age-dependent sex difference in tDCS current intensity resulting from cerebral atrophy (Bhattacharjee et al., 2022).

The BDNF Val66Met polymorphism status of participants in the research interacted with the tDCS application, resulting in increased MD in the Rt. UF and decreased MD and RD in the Lt. cingulum of the cingulate cortex of the Met non-carriers after sequential tDCS. The BDNF has been shown to affect white matter microstructure by modulating myelinogenesis (Du et al., 2003), but its impact varies depending on factors such as age and type of tract (Voineskos et al., 2011; Tost et al., 2013). A previous study found that the BDNF Val66Met polymorphism interacts with age to affect white matter microstructure, particularly in corticocortical association tracts and late-myelinating fiber tracts in the brain, suggesting that older Val66 homozygotes may be more susceptible to changes in white matter microstructure (Voineskos et al., 2011). However, in the present study, the Rt. UF and Lt. cingulum of the cingulate cortex showed differential changes after tDCS application in Val66 homozygotes, despite both being the latest-myelinated fibers. Furthermore, changes in the microstructural integrity of the Rt. UF were found to be different between *APOE* ε4 carriers and older Val66 homozygotes, although both groups are susceptible to AD. Relative to *APOE* ε4 carrier status, the BDNF Val66Met polymorphism has shown inconsistent effects on white matter integrity, with both increased and decreased FA and RD reported in different brain tracts (Soliman et al., 2010; Chiang et al., 2011; Tost et al., 2013). This highlights the complexity of the interaction between BDNF Val66Met polymorphisms, white matter integrity, and the response to tDCS. Further research is therefore needed into the factors that may contribute to this inconsistency.

This study also found that tDCS interacts with multiple factors to affect white matter integrity who were Val66 homozygotes and *APOE* ε4 carriers following sequential tDCS. Similarly, female with Val66 homozygosity showed a significant improvement in white matter integrity in the Rt. UF after tDCS sessions. Previous studies have shown accelerated Aβ deposition in *APOE* ε4 carriers with BDNF Met carriers (Stonnington et al., 2020; Riphagen et al., 2022), but there is limited investigation into the impact on the white matter integrity. Regarding the sex, although some studies suggest a sexually dimorphic effect of the BDNF Met66 allele on AD susceptibility (Fukumoto et al., 2010), others contradict this (Voineskos et al., 2011). In the prodromal phase of AD, Val66 homozygotes with a higher susceptibility to age-related decline in white matter integrity (Voineskos et al., 2011) are assumed to induce a greater increase in integrity in the AD susceptible tract in response to sequential tDCS in the presence of AD risk factors. In addition, the UF that shows an interaction between tDCS and AD risk factors in this study is a pathway that connects the anterior temporal lobe to the orbitofrontal cortex and is involved in cognitive functions such as episodic memory and language (Von Der Heide et al., 2013). It is therefore worth investigating how improved integrity in this pathway affects the clinical course of AD in the long term.

In this investigation, we observed a significant correlation between baseline Aβ deposition and enhanced FA in the left cingulum within the hippocampal region, following the administration of sequential tDCS. This aligns with prior research that reported diminished fiber connections in this specific area among patients in the early stages of AD and those with MCI (Zhou et al., 2008). Moreover, we identified a positive association between the increase in FA of the Lt. cingulum in the hippocampal region and improvements in delayed memory performance, corroborating earlier findings (Zhou et al., 2008). In subgroups stratified by AD risk factors, we also found a positive association between Aβ deposition and the difference in FA of the left cingulum in the hippocampal area in *APOE* ε4 carriers. However, regarding the FA of this tract, there was not significant relationship with the differences in neuropsychological performances. Taken together, these observations suggest a potential compensatory mechanism whereby Aβ deposition triggers alterations in the white matter integrity of the Lt. cingulum in the hippocampal area, especially in the *APOE* ε4 carriers. This change could be catalyzed by the application of sequential tDCS during the prodromal phase of AD. Nonetheless, further research with larger sample size is warranted to substantiate these preliminary insights.

A limitation of this study is the duration of sequential tDCS. As this study only involved a 10-session application of tDCS, conducting a long-term study would offer a more comprehensive understanding of the clinical implications of the compensatory changes in white matter microstructure integrity that were inferred from this study. Furthermore, conducting a study that includes a sham stimulation group would enhance our understanding of the role and clinical implications of the effect modifiers identified in the present study with MCI patients.

The purpose of this research was to assess the impact of a 2-week application of sequential tDCS on changes in white-matter microstructure integrity of MCI patients, taking into account individual factors for AD. Additionally, we examined the association between baseline Aβ deposition and changes in white matter microstructure integrity, as well as the relationship between changes in cognitive function and those in white matter integrity. The study revealed a significant effect of the effect modifiers*tDCS interaction on the white matter microstructure integrity of the AD vulnerable tract. Furthermore, there was a positive association between Aβ deposits and changes in the integrity of the white matter tract which reflects the AD progression. Finally, the changes in the tract of interest also exhibited a positive relationship with the differences in the memory performance in the prodromal stage of AD. AD is a multi-faceted neurodegenerative disease that elicits various responses to treatments among patients diagnosed with MCI. Therefore, it is essential to apply a precision medicine therapeutic approach that takes into account individual AD-related factors, especially regarding non-invasive brain stimulation. In this regard, this study provides a cornerstone for the clinical importance of precision medicine approaches in tDCS and in the field of non-invasive brain stimulation therapy for patients on the AD trajectory. Additionally, conducting additional research to address the limitations of the current study would provide an opportunity to reassess the therapeutic potential of tDCS in the prodromal AD phase, which has limited treatment options available.

## Data availability statement

The datasets presented in this article are not readily available because the datasets generated or analyzed during the current study are not publicly available due to the Patient Data Management Protocol of Yeouido Saint Mary’s Hospital but are available from the corresponding author upon reasonable request. Requests to access the datasets should be directed to DWK, kato7@hanmail.net.

## Ethics statement

The studies involving humans were approved by Institutional Review Board of the Catholic University of Korea. The studies were conducted in accordance with the local legislation and institutional requirements. Written informed consent for participation in this study was provided by the participants’ legal guardians/next of kin.

## Author contributions

DWK: conceptualization, methodology, data curation, writing–original draft, visualization, formal analysis, and funding acquisition. S-MW: methodology, data curation, and writing–review and editing. YU: software and investigation. SK: methodology and data curation. TK: methodology and data curation. DK: methodology and data curation. CL: conceptualization and supervision. HL: conceptualization, methodology, writing–review and editing, supervision, and project administration. All authors contributed to the article and approved the submitted version.

## Funding

This research was supported by the National Research Foundation of Korea (NRF) grant funded by the Korean government (Ministry of Science and ICT; No. 2019R1C1C1007608) and a grant of the Korea Health Technology R&D Project through the Korea Health Industry Development Institute (KHIDI), funded by the Ministry of Health & Welfare, Republic of Korea (grant number: HI22C0467).

## Conflict of interest

HL, TK, and DK were employed by NEUROPHET Inc.

The remaining authors declare that the research was conducted in the absence of any commercial or financial relationships that could be construed as a potential conflict of interest. The data processing services provided by NEUROPHET Inc. were utilized to enhance the quality and analysis of the brain imaging data collected during the study. Authors declare that the research outcomes and conclusions remain unbiased and are not influenced by any commercial interests associated with the NEUROPHET Inc.’s products or services.

## Publisher’s note

All claims expressed in this article are solely those of the authors and do not necessarily represent those of their affiliated organizations, or those of the publisher, the editors and the reviewers. Any product that may be evaluated in this article, or claim that may be made by its manufacturer, is not guaranteed or endorsed by the publisher.

## Footnotes

^1^https://www.fil.ion.ucl.ac.uk/spm/software/spm12

^2^https://www.nitrc.org/projects/panda/

^3^https://fsl.fmrib.ox.ac.uk/fsl/fslwiki/FSL

^4^https://www.jamovi.org
